# Adaptation strategies of iron-oxidizing bacteria *Gallionella* and Zetaproteobacteria crossing the marine–freshwater barrier

**DOI:** 10.1128/mbio.02572-24

**Published:** 2025-09-23

**Authors:** Petra Hribovšek, Emily Olesin Denny, Achim Mall, Håkon Dahle, Ida Helene Steen, Runar Stokke

**Affiliations:** 1 Centre for Deep Sea Research, University of Bergen 1658, Bergen, Norway; 2 Department of Earth Science, University of Bergen 1658, Bergen, Norway; 3 Department of Biological Sciences, University of Bergen 1658, Bergen, Norway; 4 Computational Biology Unit, University of Bergen 1658, Bergen, Norway; University of Vienna, Vienna, Austria

**Keywords:** *Gallionella*, *Mariprofundus*, Zetaproteobacteria, iron oxidation, hydrothermal vents, marine, freshwater

## Abstract

Iron-oxidizing Betaproteobacteria and Zetaproteobacteria are generally associated with freshwater and marine environments, respectively. Despite repeated cross-environment observations of these taxa, there has been no focused exploration of the genomes of marine *Gallionella* (Betaproteobacteria) to understand transitions between freshwater and marine habitats. Consequently, their roles in these environments remain uncertain. Here, we present strong evidence for the co-occurrence of *Gallionella* and Zetaproteobacteria at deep-sea hydrothermal vents at the Arctic Mid-Ocean Ridges through metagenomic analyses. Phylogenomic analysis of *Gallionella* metagenome-assembled genomes (MAGs) suggests that seawater adaptation is an evolutionary event that occurred multiple times in distinct lineages. Similarly, several distinct evolutionary events for freshwater and terrestrial *Mariprofundus* and other Zetaproteobacteria are predicted. The presence of *c*-type cytochrome *cyc2* iron oxidation genes in co-occurring marine Betaproteobacteria and Zetaproteobacteria implies an overlap in niches of these iron-oxidizers. Functional enrichment analyses reveal genetic differences between marine MAGs of both iron-oxidizing groups and their terrestrial aquatic counterparts linked to salinity adaptation. Though scanning electron microscopy confirms the presence of Fe(III) oxyhydroxide stalks where *Gallionella* and *Mariprofundus* co-occur, *Gallionella* MAGs from hydrothermal vents lack evidence of putative stalk formation genes. *Mariprofundus* is therefore the likely sole stalk-producing iron-oxidizer in this environment. Conversely, the discovery of putative stalk formation genes in *Mariprofundus* MAGs across the marine-freshwater barrier suggests that Fe(III) oxyhydroxide stalks might not be an exclusive signature for single iron-oxidizing taxa in marine and freshwater environments. Our research provides novel insights into the iron-oxidizing capacities, stalk production, environmental adaptation, and evolutionary transitions between marine and freshwater habitats for *Gallionella* and Zetaproteobacteria.

**IMPORTANCE:**

Iron-oxidizing bacteria (FeOB) play an important role in the global cycling of iron, carbon, and other metals. While it has previously been assumed that bacterial evolution does not frequently involve crossing the salinity barrier, recent studies indicate that such occurrences are more common than previously thought. Our study offers strong evidence that this also happens among FeOB, with new insights into how these bacteria adapt to the new environment, including hydrothermal vents and freshwater habitats. In addition, we emphasize the importance of accurate iron-oxidizing taxa identification through sequencing, rather than relying solely on the morphology of Fe(III) oxyhydroxides and environment. On a larger scale, microorganisms within established communities need to respond to changes in salinity due to events like seawater intrusion in coastal aquifers, and thus, our findings underscore the importance of knowledge of transitions across habitat types with different salt concentrations.

## INTRODUCTION

Salinity is reported as the major factor shaping bacterial community composition between marine and freshwater environments (1). This is supported by numerous studies on communities along salinity gradients (2–5). Microbial marine–freshwater transitions are thought to be infrequent events, with marine and freshwater communities displaying differences in the abundance of major phyla and habitat-specific lineages (6). Conversely, many taxa are reproducibly observed in both environments, often at low abundances, supporting the idea that transitions between marine and freshwater ecosystems occur more frequently than anticipated (7). Several studies underscore the importance of knowledge of transitions across habitat types due to evidence of altered metabolic capacity of microbial communities across salinity gradients (8, 9). How taxa fulfilling critical ecosystem functions respond across marine and freshwater systems is key to document as the salinity of water bodies continues to shift with changes in Earth climate regimes and anthropogenic disruption (10–12).

Organisms that move from freshwater to marine environments are exposed to osmotic stress caused by increased salt concentration in the environment. To adapt to osmotic pressure, marine microorganisms use strategies such as accumulation of ions using ion channels (13, 14) and import or production of compatible solutes intracellularly (15, 16). Transition to marine habitats involves acquiring genes linked to osmoregulation via horizontal gene transfer (9, 17, 18), often from marine bacteria (19, 20). In comparison to freshwater and terrestrial environments, microorganisms colonizing marine environments have been found to have smaller genomes (21, 22) and more acidic proteins (23–25).

Iron-oxidizing bacteria (FeOB) within the Betaproteobacteria family Gallionellaceae and the class Zetaproteobacteria are two groups for which the genomic consequences of freshwater-marine transitions are unreported. FeOB play an important role in the cycling of iron and other elements (26, 27) and heavy metal transport (28). FeOB are early colonizers of metal surfaces and promote corrosion (29, 30). Neutrophilic microaerophilic FeOB belonging to Betaproteobacteria within the family Gallionellaceae are usually observed in freshwater and other terrestrial environments (31–38). Before the discovery of Zetaproteobacteria, Fe(III) oxyhydroxide structures at deep-sea hydrothermal vents were mistaken to be produced by freshwater FeOB like *Gallionella* (39, 40). Iron-oxidizing Betaproteobacteria genera like *Gallionella*, *Leptothrix*, and *Sideroxydans* (now placed under Betaproteobacteria within NCBI and Gammaproteobacteria within Genome Taxonomy Database [GTDB]) are presently considered as limited to low-salinity environments and generally described as freshwater genera, while Zetaproteobacteria are described as a marine class (41). The present consensus is that Fe(III) oxyhydroxide stalks in freshwater can be attributed to Betaproteobacteria (*Gallionella*, *Ferriphaselus*) (42–46) and to Zetaproteobacteria (*Mariprofundus*) in marine environments (47, 48).

*Gallionella* has also been observed at some marine and brackish-associated sites (3, 49–53). 16S rRNA gene fragments of iron-oxidizing genera of Gallionellaceae were also detected at deep-sea hydrothermal vents (54–61), with Zetaproteobacteria and Gallionellaceae FeOB often co-occurring. Similarly, Zetaproteobacteria are not only present at marine hydrothermal vents, but have also been found in coastal and brackish sediments (52, 62–70), microbial mats in estuarine environments (3), water columns of stratified estuaries (71, 72), and terrestrial aquatic environments (73–75), where they sometimes co-occur with *Gallionella* and other Gallionellaceae (3, 28, 50, 76–83). Despite these observations of FeOB outside of their typical environments, their genomes have not been explored specifically to comprehend transitions between freshwater and marine habitats and their role in these environments remains unclear.

In this study, we present newly reconstructed metagenome-assembled genomes (MAGs) of *Gallionella* and *Mariprofundus* from hydrothermal vents. We show the presence of *Gallionella* at 4% relative abundance at diffuse venting at the Arctic Mid-Ocean Ridges, where scanning electron microscopy (SEM) confirmed the presence of Fe(III) oxyhydroxide stalks, where both *Gallionella* and *Mariprofundus* were detected. The co-occurrence of the two genera also implies an overlap in iron-oxidizing niches between Betaproteobacteria and Zetaproteobacteria. Our study gains insights into the iron-oxidizing capabilities, stalk production, adaptation to marine and freshwater environments, and the evolutionary transition between these habitats for FeOB genera *Gallionella* and *Mariprofundus*. Through genus-level pangenomes and functional enrichment analyses, we uncover differences in osmoprotectant biosynthesis and transporter genes, as well as specific trends in genome size, influencing the diversification of *Gallionella* and *Mariprofundus* across the marine-freshwater divide.

## RESULTS

### *Gallionella* and Zetaproteobacteria co-occur at hydrothermal vents

**Fig 1:**
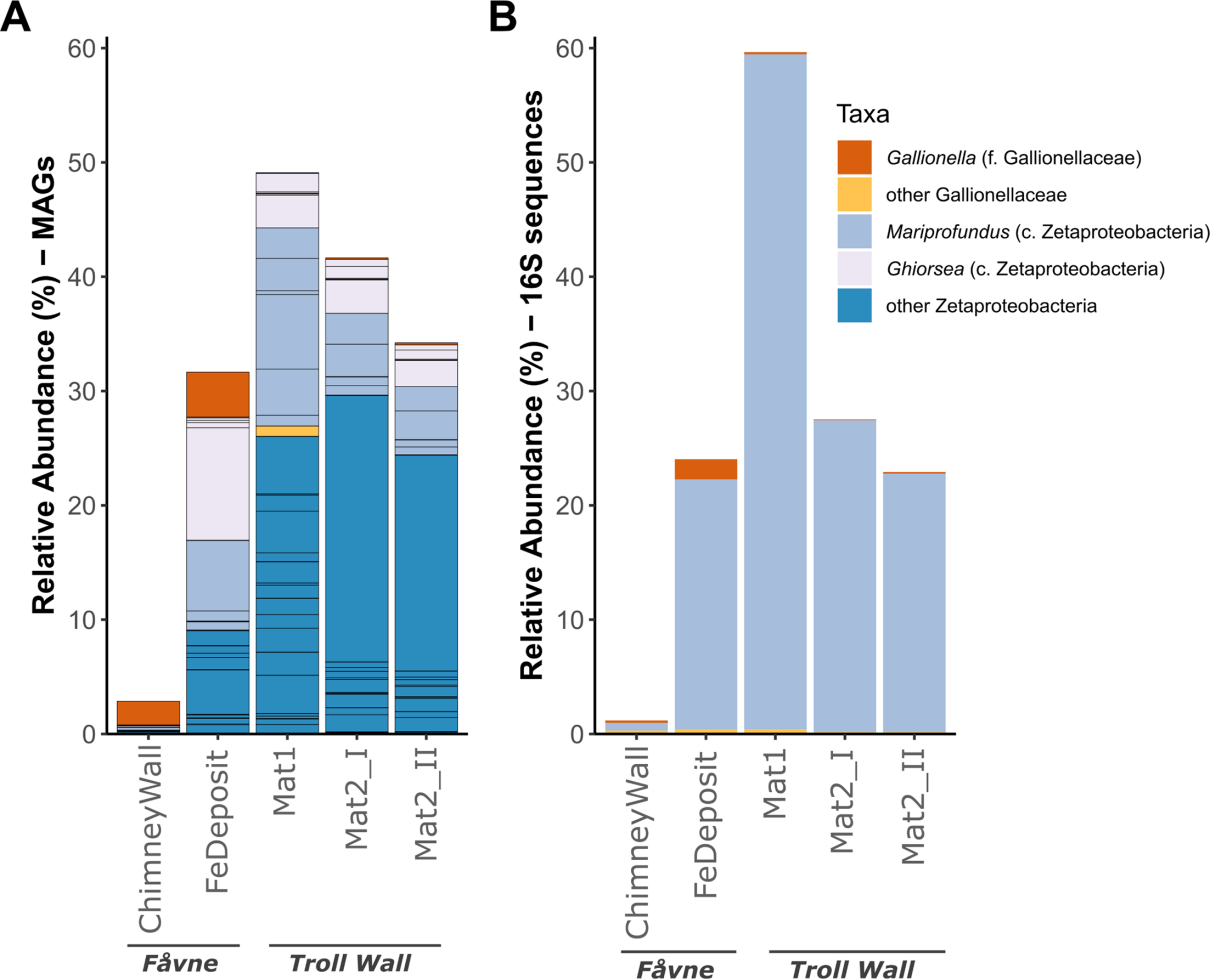
Diversity and relative abundance of FeOB at hydrothermal vents where *Gallionella* is detected at Fåvne and Troll Wall vent fields. (**A**) Relative abundance of FeOB MAGs belonging to Gallionellaceae and Zetaproteobacteria. Relative abundances are based on reconstructed MAG coverage representing the percentage of the total coverage of all detected MAGs in the sample. Black lines demonstrate the presence of several different MAGs within a taxonomic group, dereplicated at 98% ANI. (**B**) Relative abundance of FeOB belonging to Gallionellaceae and Zetaproteobacteria based on all 16S sequences from the whole metagenome (PhyloFLASH).

**Fig 2:**
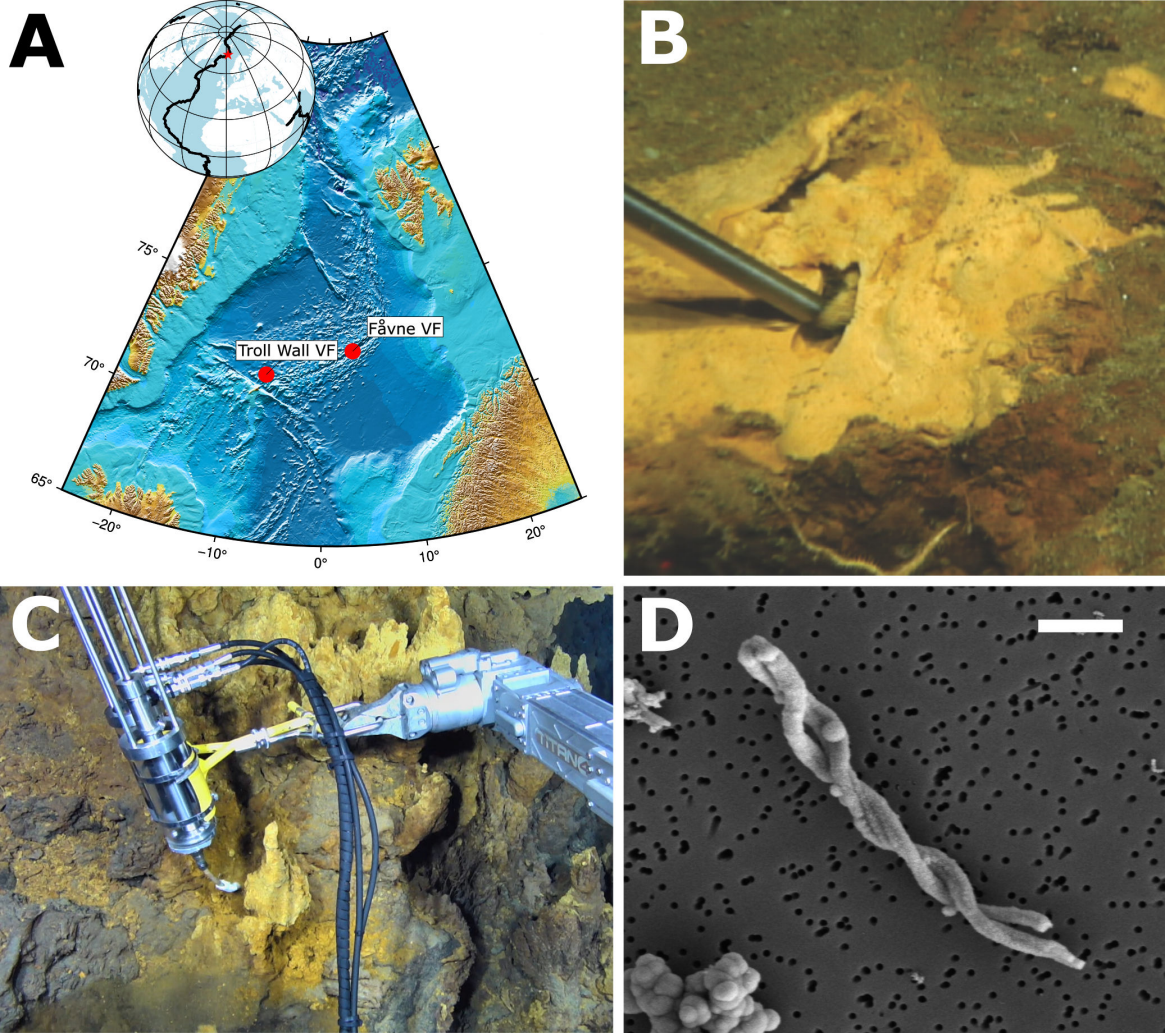
Hydrothermal vent sites where *Mariprofundus* and *Gallionella* co-occur. (**A**) Location of hydrothermal vent sites in the Arctic from where both *Mariprofundus* and *Gallionella* MAGs were reconstructed. Map generated using GMT and IBCAO grid. (**B**) Seafloor Fe mat at the Troll Wall vent field (Jan Mayen vent fields, Mat2 sample). Image modified from Vander Roost et al. (84) under Creative Commons Attribution License (55). (**C**) Sampling of diffuse venting iron oxide deposit (~10°C) at Fåvne vent field. (**D**) Scanning electron microscopy showing biogenic Fe(III) oxyhydroxide stalks in iron oxide deposit at Fåvne, where both *Mariprofundus* and *Gallionella* MAGs were present. The scale represents 2 µm.

MAGs representing two novel species or populations of *Gallionella* were reconstructed from iron oxide deposits, outer chimney wall, and microbial iron mats (Fe mats) at diffuse hydrothermal venting sites at the Fåvne and Troll Wall vent fields (Fig. 1 and 2A through C; see Tables S1 and S2 at https://doi.org/10.5281/zenodo.16565895). A single *Gallionella* MAG (AMOR20_M0989) was present with a coverage representing 4% of the total coverage of all detected MAGs in an iron oxide deposit at Fåvne (~10°C), while another *Gallionella* MAG (AMOR20_M0988) is present at 2% of the binned population in a chimney wall at Fåvne. Within these samples with highly abundant (1–4%) *Gallionella* MAGs, the iron oxide deposit also harbors several Zetaproteobacteria taxa (two families and six defined genera as classified by GTDB) comprising 28% of the binned community altogether, while the chimney wall sample has co-occurring Zetaproteobacteria with 1% relative abundance (Fig. 1A; see Table S4 at https://doi.org/10.5281/zenodo.16565895). From the same metagenomes, 15 novel species-representative genomes of Zetaproteobacteria including several within genera *Mariprofundus* and *Ghiorsea* were recovered based on ANI analyses (95% cutoff) using publicly available MAGs (see Table S3 at https://doi.org/10.5281/zenodo.16565895).

*Gallionella* MAGs are present at lower abundances at Troll Wall in comparison to Fåvne. In the Troll Wall vent Fe mats (between 2.5 and 5°C), where a Gallionellaceae MAG is present at 1% (see Table S5 at https://doi.org/10.5281/zenodo.16565895), Zetaproteobacteria MAGs are present at 48% of the binned community. When considering MAGs with coverage values above 0.5, *Gallionella* MAGs co-occur with Zetaproteobacteria in all samples taken from the Fåvne and Troll Wall vent fields (see Table S4 at https://doi.org/10.5281/zenodo.16565895). The diversity of FeOB Gallionellaceae is lower than for Zetaproteobacteria, also at the genus level (Fig. 1).

**Fig 3:**
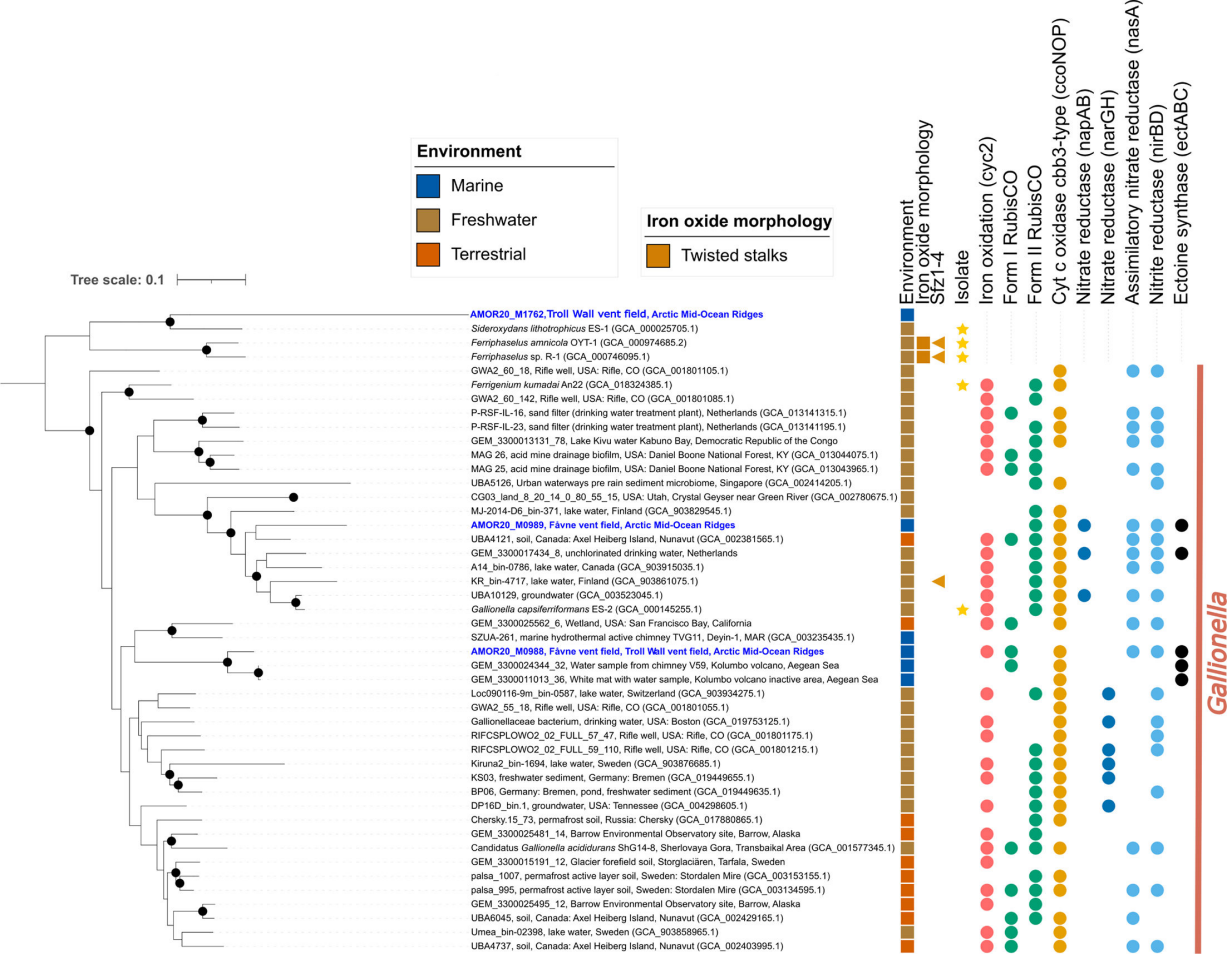
Phylogeny of Betaproteobacteria within the genus *Gallionella*. The phylogenomics tree is based on a concatenated alignment of a manually curated set of 15 single-copy gene markers (see Table S6 at https://doi.org/10.5281/zenodo.10670305) using MAGs from this study and references. All MAGs represent a species-level cluster (using 95% ANI cutoff), except for marine *Gallionella* MAGs, which were not clustered. Sfz1-4: potential stalk formation genes. Environmental assignments are based on NCBI metadata. Completeness and contamination values for each MAG are based on CheckM2 predictions. Genomes reconstructed from the Fåvne vent field and Troll Wall vent field are shown in blue. The maximum likelihood tree was constructed with IQTREE using substitution model GTR20+F+R6. Black node circles mark branches with support values higher than 80% with SH-like approximate likelihood ratio test and 95% with ultrafast bootstrapping, both including 1,000 iterations. The tree is rooted using non-*Gallionella* Gallionellaceae MAG sequences as an outgroup.

**Fig 4:**
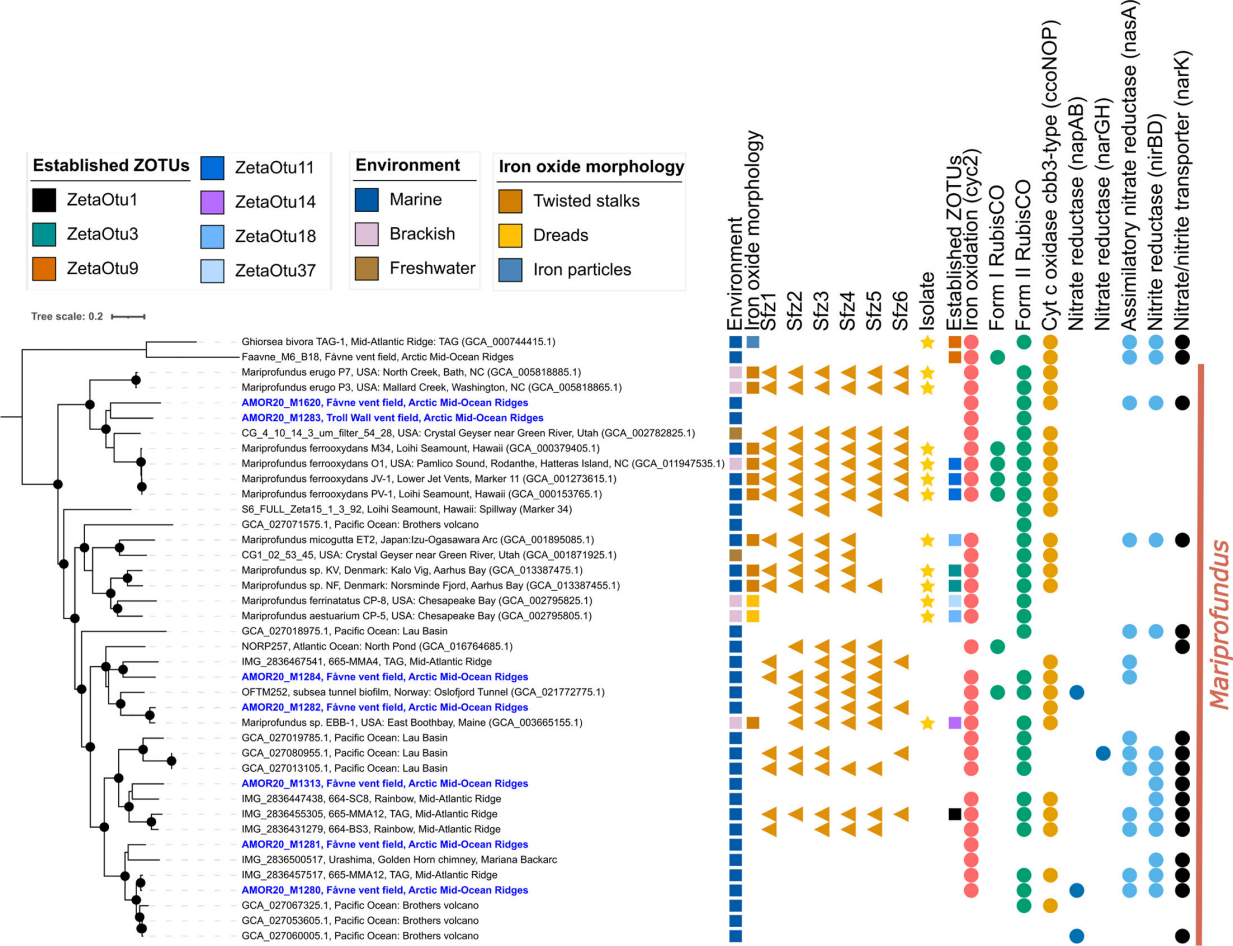
Phylogeny of Zetaproteobacteria within the genus *Mariprofundus*. The phylogenomics tree is based on a concatenated alignment of a manually curated set of 12 single-copy gene markers (85) using MAGs from this study and references. Sfz1-6: potential stalk formation genes in Zetaproteobacteria. The environments specified are based on NCBI metadata. Genomes shown in blue have been reconstructed from the Fåvne vent field and Troll Wall vent field. The maximum likelihood tree was generated using IQTREE with the substitution model LG+F+R9. Black node circles mark branches with support values higher than 80% with SH-like approximate likelihood ratio test and 95% with ultrafast bootstrapping, both including 1,000 iterations. The root of the tree is based on *Ghiorsea* MAG sequences as an outgroup.

SEM confirms the presence of Fe(III) oxyhydroxide stalks in the iron oxide deposit sample where both *Gallionella* and *Mariprofundus* MAGs were detected (Fig. 2D). In *Gallionella* MAGs from Fåvne and Troll Wall vent fields, genes potentially needed for stalk formation (*sfz1-4*) were not identified (Fig. 3). *Gallionella* MAGs sourced from other marine sites follow a similar trend. As a follow-up to this finding, an assessment of all Zetaproteobacteria MAGs stalk formation potential revealed *Mariprofundus* MAGs from freshwater environments do possess putative stalk formation genes (Fig. 4).

### Marine *Gallionella* are closely related to their freshwater counterparts

To assess whether marine *Gallionella* populations are closely related to freshwater *Gallionella*, we performed phylogenomic analysis (Fig. 3). The two recovered deep-sea hydrothermal vent *Gallionella* MAGs from Fåvne and Troll Wall are AMOR20_M0989 (97.1% completeness and 0.1% contamination), with the closest relative from permafrost (GEM_3300012005_15, 74.8% ANI), and AMOR20_M0988 (93.8% completeness and 4.9% contamination), with closest relative from a hydrothermal system of Kolumbo volcano (GEM_3300011013_36, 84.4% ANI). *Gallionella* genome SZUA-261 from active and inactive marine hydrothermal chimneys at the Deyin-1 vent on the Mid-Atlantic Ridge (57) is different from both AMOR *Gallionella* MAGs at 70% ANI. Phylogenomic analysis using 15 manually curated marker genes suggests two separate evolutionary events for marine *Gallionella*, which cluster together with their freshwater and terrestrial counterparts (Fig. 3, see Fig. S1 at https://doi.org/10.5281/zenodo.16565895). The presence/absence dendrogram in our pangenome also shows two different marine *Gallionella* clusters. In contrast, phylogenomic analysis using different sets of markers shows either three or two clusters of the marine *Gallionella* (see Fig. S2 and S3 at https://doi.org/10.5281/zenodo.16565895). Four marine *Gallionella* MAGs are unique species-level representative genomes, with two MAGs from the subsea Kolumbo volcano sharing more than 98% ANI similarity.

### Marine Zetaproteobacteria are closely related to their freshwater counterparts

To assess the extent to which close evolutionary relationships can be found between Zetaproteobacteria in marine and freshwater environments, phylogenomics, including all AMOR and publicly available Zetaproteobacteria MAGs, was performed. Genome-resolved metagenomics of samples from the Troll Wall vent field resulted in 16 Zetaproteobacteria MAGs in addition to the already published MAGs from Fe mats on Fåvne vent field black smokers (85) (see Table S3 at https://doi.org/10.5281/zenodo.16565895). Phylogenomic analysis revealed two distinct clusters of freshwater *Mariprofundus* grouping among marine representatives (Fig. 4). Similarly, several distinct clusters of hot spring Zetaproteobacteria were clustering together with their marine counterparts (see Fig. S4 at https://doi.org/10.5281/zenodo.16565895), suggesting adaptation of marine Zetaproteobacteria to freshwater conditions is an evolutionary event that has occurred multiple times in distinct lineages.

### Iron and carbon metabolism is conserved in *Gallionella* in the marine environment

Analysis of iron oxidation genes shows a conserved Fe energy metabolism in marine *Gallionella* at the Fåvne and Troll Wall vent fields. One of the *Gallionella* MAGs from a marine cluster encodes a *cyc2* gene, while the marine *Gallionella* MAG that is outside this cluster does not show the presence of *cyc2* genes. However, these genes can be found in closely related freshwater and terrestrial MAGs (Fig. 3). Incomplete aerobic and anaerobic respiratory pathways are present in marine *Gallionella*, as well as genes involved in CO_2_ fixation. A cluster of several marine *Gallionella* MAGs encodes a Form I Rubisco adapted to higher O_2_ concentrations, while the individual marine *Gallionella* MAG encodes a Form II Rubisco adapted to low oxygen concentrations (86, 87). Marine *Gallionella* MAGs also contain genes for *cbb_3_*-type cytochrome oxidase; an oxidase better adapted to low oxygen conditions (88). To further assess the ability of *Gallionella* and Zetaproteobacteria to oxidize iron outside their typical environments, MAGs from various global locations were included. Iron oxidation genes are present in FeOB genomes across the marine-freshwater barrier, with *cyc2* also present in freshwater Zetaproteobacteria MAGs (Fig. 4; see Fig. S4 at https://doi.org/10.5281/zenodo.16565895). No uptake hydrogenases indicating hydrogen as a putative alternative electron donor were detected in *Gallionella* MAGs from Fåvne vent field or other marine environments.

### Environmental adaptation in marine *Gallionella* genomes

Functionally enriched genes in marine *Gallionella* are associated with environmental adaptation, such as salinity adaptation using antiporters and osmolyte production, heavy metal resistance, resistance against viral attack and other organisms, uptake of potential nutrients or antiporters, transport systems, and membrane-bound proteins (see Table S7 at https://doi.org/10.5281/zenodo.16565895). Sulfatase genes are enriched and present in thre MAGs of marine *Gallionella*. Enriched genes in marine *Gallionella* MAGs encode for biosynthesis of osmolyte ectoine (89) and biosynthesis of nine-membered enediyne core, known for antibiotic and antitumoral activities (90). Genes involved in trehalose biosynthesis are enriched in terrestrial MAGs. Genes for potassium transporter are enriched in freshwater and terrestrial *Gallionella* MAGs (more than 80% of MAGs), while genes for sodium/alanine symporter, sodium/proline symporter, sodium/dicarboxylate symporter, and sodium/hydrogen antiporter are enriched in marine *Gallionella* MAGs.

**Fig 5:**
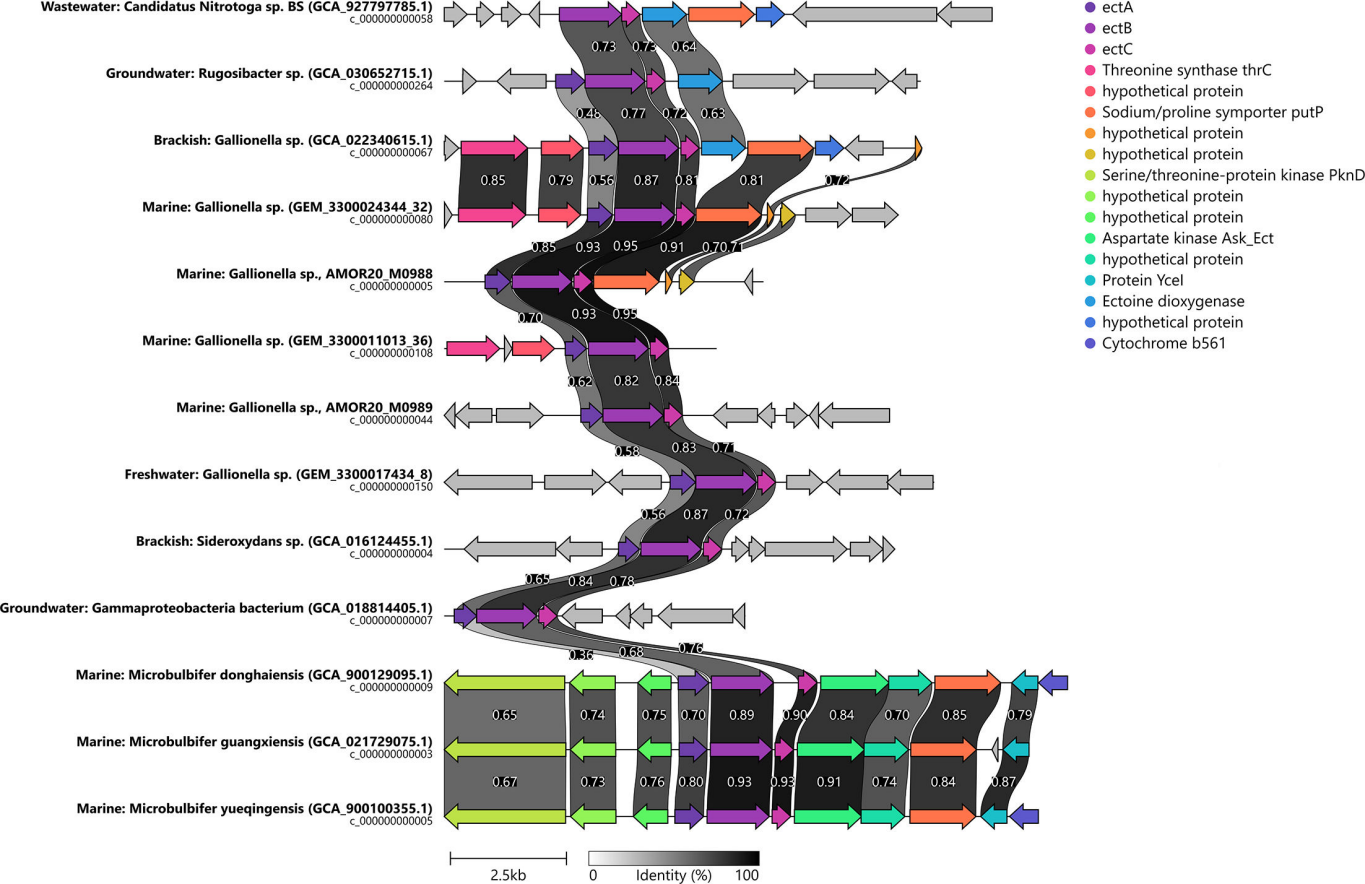
Gene neighborhood of ectoine synthase genes present in *Gallionella* MAGs including gene sequences from all publicly available *Gallionella* genomes and closely related and publicly available reference genomes. Ectoine synthase genes (ectA, ectB, and ectC) and flanking genes are highlighted by colors as in the legend.

The ectoine synthase gene was present in a majority of marine *Gallionella* (four out of five MAGs) (Fig. 3 and 5). Exploring the potential acquisition of ectoine synthase genes through horizontal gene transfer, BLAST searches and phylogenetic tree reconstruction pinpoints potential sources such as other Gallionellaceae members, unknown Gammaproteobacteria, and Gammaproteobacteria of genus *Rugosibacter* and *Microbulbifer,* commonly present in marine sediments (91) (Fig. 5; see Fig. S5 at https://doi.org/10.5281/zenodo.16565895). Closest ectoine synthase gene relatives to marine *Gallionella* within Gallionellaceae are *Gallionella* and *Sideroxydans* from brackish environments and a non-iron oxidizing *Nitrotoga* sp. BS from a wastewater treatment sample tolerant to up to 1% salt (92). Within the *Gallionella* genus, ectoine synthase genes exhibit 80–85% identity and 60–65% similarity to genes of *Microbulbifer* found in marine and brackish environments. Among these genes, *ectB* and *ectC* demonstrate a more conserved trend compared to *ectA* (Fig. 5).

### Environmental adaptation and nitrate assimilation in Zetaproteobacteria genomes

Genes for potassium/hydrogen antiporter that regulate potassium levels within cells, preventing toxicity in low osmolarity situations (93, 94) are enriched in more than 66% of freshwater *Mariprofundus* MAGs and ca 10% marine MAGs, while genes for sodium/hydrogen antiporter are enriched in brackish and marine Zetaproteobacteria MAGs. Moreover, genes for sodium or hydrogen/acetate symporter are enriched both in marine and freshwater Zetaproteobacteria MAGs. Twenty-five percent of Zetaproteobacteria MAGs were assigned as brackish (eight MAGs) and a few marine MAGs possess genes responsible for producing osmoprotectants like trehalose, a spermidine/putrescine transport system, acetoin utilization protein, and glutamate/leucine dehydrogenase. *NarK* gene encoding for a nitrate/nitrite transporter (95, 96) possibly involved in nitrate assimilation is exclusively present in 58% of marine MAGs (see Table S8 at https://doi.org/10.5281/zenodo.16565895). A similar trend is observed in the genus *Mariprofundus* (see Table S9 at https://doi.org/10.5281/zenodo.16565895). Mercury resistance gene (*merR1*) is found in 13 Zetaproteobacteria MAGs exclusively from hydrothermal vents (see Fig. S4 at https://doi.org/10.5281/zenodo.16565895).

### Further outlook into genomic adaptations

**Fig 6:**
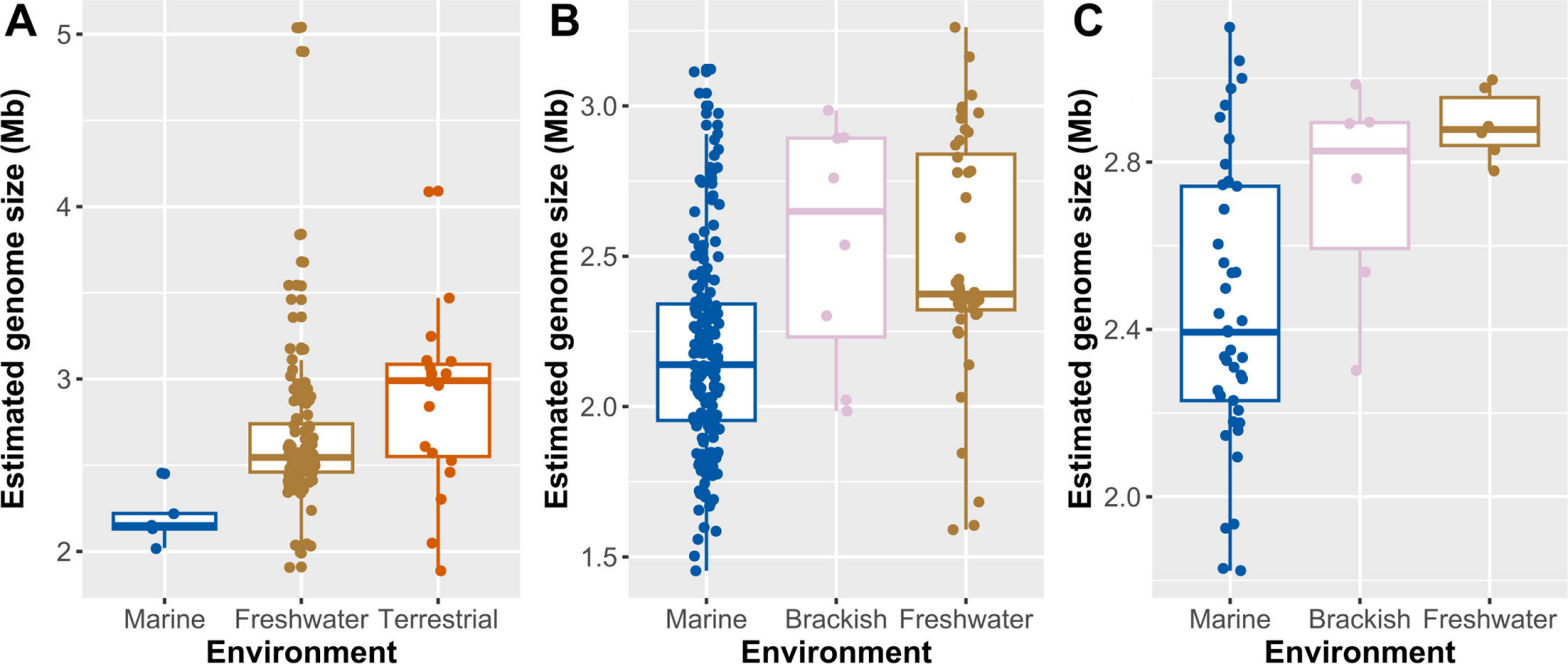
Estimated genome sizes of *Gallionella* and Zetaproteobacteria genomes from marine, brackish, freshwater, and terrestrial environments. Publicly available genomes and MAGs from this study. Boxplots visualize the distribution of estimated genome sizes for each environment, showing mean and standard deviation with points visualizing the estimated genome sizes of all individual MAGs. (**A**) *Gallionella* MAGs. (**B**) Zetaproteobacteria MAGs. (**C**) *Mariprofundus* MAGs.

Generally, small average genome size (2.19 ± 0.16 Mb), high percentage of coding density (91% ± 2.1%) and low GC content (49.7% ± 2.5%) are observed in marine *Gallionella* MAGs (see Table S10 at https://doi.org/10.5281/zenodo.16565895). Performing both the one-way ANOVA and the Kruskal-Wallis test with appropriate *post hoc* tests to examine the effects of the environment on genome size, differences were detected between marine and terrestrial *Gallionella* genomes. Assessment of Zetaproteobacteria genome sizes, previously limited to marine environments where genome streamlining within the class was suggested (97), revealed that marine genomes of Zetaproteobacteria are smaller compared to their brackish and freshwater counterparts (Fig. 6; see Table S10 at https://doi.org/10.5281/zenodo.16565895). Marine *Mariprofundus* genomes are smaller than freshwater ones. Genome size varies greatly within environmental groups, however, and due to few genomes in some groups, such as the marine *Gallionella* genomes, the power analysis indicates that sampling of more genomes is required to be confident in the conclusion at the significance level of 0.05.

Whole-proteome isoelectric point (pI) comparison of *Gallionella* predicts proteomes exhibit mostly similarities across the marine-freshwater barrier (see Fig. S6 at https://doi.org/10.5281/zenodo.16565895), while differences between environments are more obvious in Zetaproteobacteria and *Mariprofundus* (see Fig. S7 and S8 at https://doi.org/10.5281/zenodo.16565895). Average relative frequencies for acid, neutral, and basic pIs show little difference between environments (see Table S11 at https://doi.org/10.5281/zenodo.16565895). As with genome sizes, sampling of more genomes is required for statistical analyses with great confidence.

## DISCUSSION

This comparative genomics study investigated how members of freshwater *Gallionella* may adapt to marine conditions and how marine Zetaproteobacteria may adapt to freshwater conditions. Our study includes the reconstruction of novel *Gallionella* MAGs from diffuse venting at marine hydrothermal vents, providing further evidence that the adaptive transition from a freshwater setting to a marine setting, or vice versa, can occur over relatively short evolutionary timescales among *Gallionella*. Here, we consider aspects of the transition of the marine-freshwater barrier and offer a model involving adaptations of FeOB to higher salinity via horizontal gene transfer and possible genome reduction.

### Autotrophic and iron-oxidizing *Gallionella* and Zetaproteobacteria co-occur in hydrothermal vents

Earlier investigations of low-salinity environments revealed the co-occurrence of Zetaproteobacteria and Gallionellaceae FeOB, in settings including brackish waters, terrestrial hot springs, benthic microbial mats, and fjord sediments (3, 28, 50, 76–83). In marine hydrothermal systems, co-occurrence of Zetaproteobacteria and Gallionellaceae FeOB was also indicated in iron mats, iron mounds, and active and inactive hydrothermal chimneys (54–58, 60). In addition to *Gallionella*, closely related iron-oxidizing Betaproteobacteria like *Sideroxydans* and *Leptothrix* have been detected with 16S rRNA gene sequences at the Troll Wall hydrothermal vent field (56).

*Gallionella* and Zetaproteobacteria MAGs recovered from the same diffuse hydrothermal vent samples at AMOR provide further evidence that co-occurrence of the two FeOB groups is globally widespread, with Zetaproteobacteria consistently present wherever *Gallionella* is detected in the marine environment. The only exception is the inactive sulfide chimneys of the East Pacific Rise, where only *Gallionella* sequences were detected (59). *Gallionella* and Zetaproteobacteria are predicted to oxidize iron regardless of environment, implying an overlap in iron-oxidizing niches between Betaproteobacteria and Zetaproteobacteria within the same community. Marine *Gallionella* present at such high abundances (up to 4% of the community) at Fåvne vent field may indicate that *Gallionella* FeOB are responsible for significant iron oxidation at diffuse venting zones of this hydrothermal field. *Gallionella* and Zetaproteobacteria at hydrothermal vents are possibly both involved in chemolithoautotrophy and processes contributing to the cycling of iron and carbon in the same environment. Interactions between these seemingly functionally redundant FeOB, such as competition, remain to be studied.

### Identity of FeOB forming Fe(III) oxyhydroxide stalks in marine and freshwater environments

Stalks of *Gallionella* and *Mariprofundus* are remarkably similar (98). Stalk-producing representatives of both Betaproteobacteria and Zetaproteobacteria were previously found to possess the *sfz* gene cluster, putatively involved in stalk formation (44, 99). Stalks and the *sfz1-4* genes (99) are suggested to be limited to the genera *Gallionella* and *Ferriphaselus* within Gallionellaceae (46). Based on the presence of both *Mariprofundus* and *Gallionella* in hydrothermal iron oxide deposits, observed stalks could theoretically be produced by either *Mariprofundus* or *Gallionella*. Even so, no evidence of putative stalk formation genes is found in *Gallionella* MAGs at hydrothermal vents (Fig. 3), whereas co-occurring *Mariprofundus* MAGs contain *sfz* genes (Fig. 4). It is therefore more likely that stalks are produced by *Mariprofundus* in this environment. *Sfz* genes do not seem widespread in *Gallionella* populations, regardless of habitat, as previously observed (46), but are rather more widely distributed in *Mariprofundus* populations. *Mariprofundus* genomes sourced from diverse environments spanning marine, brackish, and terrestrial hot springs, on the other hand, have putative stalk formation genes (Fig. 4). Consequently, Fe(III) oxyhydroxide stalks are not necessarily a definitive signature for *Mariprofundus* and *Gallionella* in marine and freshwater environments, respectively. The identity of FeOB groups has often been interpreted solely on the morphology of Fe(III) oxyhydroxides and the environment (40, 100–105). We emphasize that DNA-based identification methods are necessary for accurate FeOB identification rather than reliance on stalk morphology and environment alone. It remains unclear whether marine *Gallionella* and freshwater *Mariprofundus* express these genes and produce stalks; only microscopy and cultivation efforts will show whether this is the case. Not all *Gallionella* isolates produce stalks (45, 106, 107), neither do all *Mariprofundus* isolates (70, 99, 108). Even when a FeOB species has the ability to produce stalks, they do not appear to be essential for growth (109), as stalk formation might be affected by conditions such as cell number, Fe(II) concentrations, and pH (99, 106).

### FeOB seem to have crossed the marine-freshwater barrier in several evolutionary events

Capacity to thrive in diverse salinity conditions is proposed to have developed early in bacterial evolution (8). Although marine and freshwater bacteria co-occur in brackish environments, true microbial freshwater-marine transitions are assumed to be infrequent phenomena due to the necessity to adjust to drastic changes in physicochemical conditions. Habitat transitions may result in differences in proteomes and potentially substantial changes in central metabolism, which suggest significant evolutionary time has passed for the salinity adaptation to occur (23). General observation also supports transitional infrequency as dominant freshwater and marine taxa are usually not closely related (6). Upon close examination, it appears that certain transitioning microorganisms may in fact be present within the “rare biosphere” (7), that is, not dominant within their non-typical (marine/freshwater) environment, including *Gallionella* and Zetaproteobacteria.

Our phylogenetic analyses suggest that FeOB transitions between freshwater and marine environments could have occurred several times, with several *Gallionella* species colonizing the marine environment independently. Marine *Gallionella* genomes appear closely related to freshwater *Gallionella* (Fig. 3), confirming previous findings based on 16S rRNA gene sequences (56). As *Gallionella* seems widely present in marine environments, they appear to have successfully transitioned across the freshwater-marine boundary. Similarly, multiple crossings of the marine-freshwater barrier are observed within Zetaproteobacteria, including the genus *Mariprofundus* (Fig. 4; see Fig. S4 at https://doi.org/10.5281/zenodo.16565895). These results imply complex evolutionary histories which could be improved in resolution with more genomes and updated 16S rRNA gene sequence analyses. The direction of adaptation (fresh to saline, or vice versa) remains unclear for both groups of FeOB. Reconstructing habitat of origin for FeOB lineages might be aided through phylogenomic analyses in tandem with phylogenetic analysis of genes involved in synthesis of osmolytes, which has proved instrumental for similar investigations in other taxa (110).

The transition of FeOB between freshwater and marine environments could have been promoted by adaptations in brackish environments, as hypothesized for some other microorganisms crossing the barrier (111), though no substantial proof of such an event was detected from our phylogenomic reconstructions. In brackish microorganisms, both freshwater and marine characteristics (salt adaptation mechanisms) are present (94). *Gallionella* have ectoine synthase genes which are closely related to one another. Possible sources of the ectoine synthase genes may be through organism(s) in brackish or freshwater settings already adapted to conditions within a marine/estuarine environment or through interactions in groundwater exposed to inflow of saltwater. The combined evidence of phylogenetically similar *Gallionella* genomes across geographically spread marine and freshwater settings, likely horizontal gene transfer, and lack of pronounced differences in proteome isoelectric profiles suggests that *Gallionella* freshwater-marine transition might have been driven by a rapid rather than gradual adaptation, as suggested for several other taxonomic groups (112).

### Possible adaptations of *Gallionella* to marine environment

Comparing *Sideroxydans* and *Gallionella* based on genomic and ecological analysis, *Sideroxydans* has been seen as perhaps better adapted to conditions with higher salinity than *Gallionella* (3). In this study with observations of highly abundant marine *Gallionella* species, we now know *Gallionella* can also be adapted to higher salinity. Salinity of water masses above Mohns Ridge is expected to be 34.9 ppt—very close to the global average of 35 ppt, based on vertical CTD profile data (113). Given the high degree of dilution of hydrothermal fluids with ambient seawater upon exit at the seafloor (typically >80–90%), and the relatively limited range of salinity in most endmember vent fluids (114), most low temperature/diffuse vent fluid salinities do not differ substantially from background saltwater salinity (115). For this reason, we argue that *Gallionella* at hydrothermal vents on the AMOR is likely only exposed to salinities comparable to seawater, and we consider the possibility of some cryptic microniches of low salinity at these settings highly unlikely. This implies, therefore, that some *Gallionella* species are fully adapted to marine environments.

There are certain bioenergetic costs associated with all strategies of adaptation to higher salinity, which come with evolutionary speculations. Accumulation of salts inside the cell, though, requires the intracellular cell machinery to be adapted to higher salt concentrations (116), while this is not required by the compatible solute strategy, which may not require such a large modification to the proteome (15). However, such microorganisms need to instead invest energy in ion pumps to maintain low ionic concentrations in the cell.

**Fig 7:**
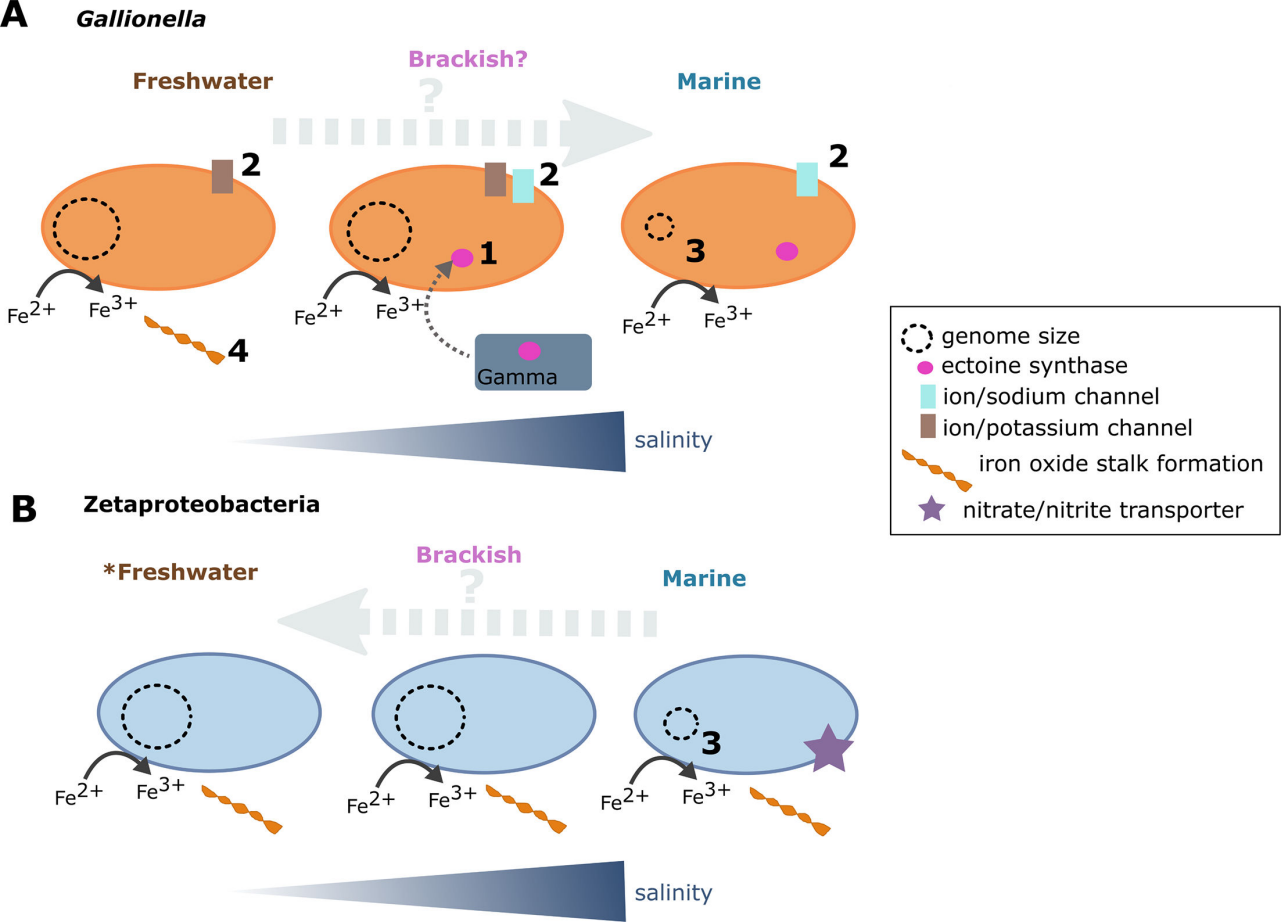
Model of adaptations in *Gallionella* (**A**) and Zetaproteobacteria (**B**) crossing the marine-freshwater barrier. The model is based on predicted genes in MAGs, involving adaptations discussed in this paper. (1) Ectoine synthase in marine *Gallionella* MAGs (possible horizontal gene transfer with Gammaproteobacteria). (2) Ion/potassium channels in freshwater microorganisms and ion/sodium channels in marine microorganisms. (3) Smaller genomes in marine microorganisms. (4) Formation of Fe(III) oxyhydroxide stalks based on putative stalk formation genes found in some representatives of *Gallionella* and Mariprofundus, however, is not in any known marine *Gallionella* MAGs. *Some MAGs are saline terrestrial, since in some terrestrial hot springs, salinity of 9–14 ppt was measured.

Salinity seems to play a role in the adaptation of marine *Gallionella* (Fig. 7). Genes for synthesis of ectoine, a compatible solute, are enriched in marine *Gallionella* genomes (Fig. 2). Genes for synthesis of compatible solutes like ectoine, possibly obtained via horizontal gene transfer, allow *Gallionella* in the marine environment to overcome osmotic stress and adapt to environments of different salinities. Production of the compatible solute ectoine as an adaptation is seen in several marine and halophilic microorganisms (89, 117–119). All marine *Gallionella* and other Gallionellaceae that have ectoine synthase genes they potentially acquired from Gammaproteobacteria. *Gallionella* genomes from different environments show differential presence of several ion pump genes that typically participate in establishing ion gradients across the cellular membrane. Freshwater organisms have been found to use ion/potassium channels, as opposed to ion/sodium channels used by marine organisms (22, 94), which was also evident in *Gallionella* MAGs. The differential adaptation to the environment with elevated salinity also involved different genes involved in lipid and fatty acid metabolism (94, 120). Although not exclusively observed in marine *Gallionella* MAGs, the production of extracellular polymeric substances might also assist in adapting to elevated salinity levels (121).

Marine *Gallionella* and Zetaproteobacteria genomes generally fall on the lower end of observed genome sizes compared to their freshwater and terrestrial counterparts, aligning with the globally observed trend across salinities (21, 22, 94). We stress that sampling of more genomes is required to statistically conclude that marine genomes of both *Gallionella* and Zetaproteobacteria are measurably smaller and/or went through genome streamlining. Distinctions in genome size are thought to arise from differences in the physicochemical environment. As freshwater habitats generally experience more rapid and pronounced fluctuations of physicochemical conditions compared to larger marine bodies of water, a larger genome of freshwater microorganisms enables them to be more flexible to adapt (19, 94).

Proteomes have previously shown more acidic values of isoelectric points (pI) in microbes exposed to marine environments and higher salinities than in freshwater and brackish microorganisms (23, 25, 94, 122, 123). Transitions between marine and freshwater environments are proposed to require long evolutionary time (23) and are difficult to attribute solely to horizontal gene transfer (1). The isoelectric point values observed in *Gallionella* predicted proteomes showed no significant variations across the marine-freshwater divide, indicating that the proteome did not undergo extensive changes in its amino acid compositions. However, values and power analysis indicate that sampling of more genomes is required to draw concrete conclusions on whether larger proteome changes were involved in *Gallionella* salinity transitions. Nevertheless, this observation raises a strong argument that *Gallionella* transitions to the marine environment did not require wider genomic adaptations but rather involved horizontal gene transfer and occurred on a shorter evolutionary time scale.

### Adaptations of Zetaproteobacteria to freshwater environments are less obvious

The presence of Zetaproteobacteria in certain terrestrial springs is possibly explained by elevated salinity levels (73), with examples of salinity between 9 and 14 ppt (79–81). The grouping may therefore not completely accurately reflect the actual physicochemical environment of the microorganisms, often excluding salinity. Due to the absence of salinity measurements for several terrestrial environments characterized and assumed as “freshwater”-associated (75, 76), it remains uncertain whether Zetaproteobacteria have the capability to inhabit “true” freshwater environments with low salinity. Patterns for genome and proteome adaptation in Zetaproteobacteria MAGs were, however, less intelligible than for *Gallionella*. Marine Zetaproteobacteria genomes group with those from seemingly freshwater environments with unknown salt concentrations, making a thorough analysis reliant on quality metadata on environmental parameters. Still, Zetaproteobacteria have shown environmental differences in osmoregulation-related adaptations such as differences in genes for compatible solutes and involved in transport. In addition, nitrate assimilation genes present exclusively in marine Zetaproteobacteria genomes (Fig. 7) suggest certain differences in nutrient uptake depending on the environment, argued to likely correspond to the typically higher concentrations of nitrate in seawater (124–126) compared to freshwater environments (41, 127, 128). Nitrogen fixation genes were observed in a few Zetaproteobacteria from both hot springs and marine environments (78, 97). Absence of nitrate and nitrite reductases in freshwater Zetaproteobacteria suggests an aerobic lifestyle, with nitrate reduction potentially not playing a big role in freshwater Zetaproteobacteria. Since hydrothermal vent fluids contain large amounts of heavy metals (129, 130) which iron oxides adsorb (131–133), FeOB at hydrothermal vents could experience more heavy metal stress than in other environments. This would lead to more heavy metal resistance genes encoded in the genomes, such as mercury resistance as observed in hydrothermal Zetaproteobacteria (see Fig. S4 at https://doi.org/10.5281/zenodo.16565895).

### Conclusion

Metagenome-resolved genomes of *Gallionella* were retrieved from deep sea hydrothermal vents*,* despite belonging to the previously recognized freshwater genus of FeOB. We provide further evidence that marine *Gallionella* can co-occur with Zetaproteobacteria FeOB and that they share an iron-oxidizing niche. Functional enrichment analyses showed several adaptations to an environment with elevated salinity. Even though only a few MAGs are available to date, our findings contribute to a better understanding of the diversification of FeOB, shedding light on their roles in the environment and their strategies for adapting to different conditions crossing the marine-freshwater barrier, including hydrothermal vents and freshwater habitats.

## MATERIALS AND METHODS

### Sample collection and processing

Samples were collected at two hydrothermal vent fields at the Arctic Mid-Ocean Ridges (AMOR). Samples were collected using an Ægir6000 remotely operating vehicle (ROV) on board the R/V G.O. SARS in July 2011, July 2012, and June 2019. The ROV was equipped with a biosyringe, a hydraulic sampling cylinder, linked to the ROV’s manipulator arm. Iron oxide deposit (FeDeposit) and outer chimney wall (ChimneyWall) were collected at Fåvne vent field (85, 134–136) at approx. 3,036 m below sea level (Fig. 2C; see Table S1 at https://doi.org/10.5281/zenodo.16565895). Fe mat samples (Mat1, Mat2_I, and Mat2_II) were collected from the Rift valley at Troll Wall vent field, one of Jan Mayen vent fields (55, 56, 84) at approx. 615 m below sea level (Fig. 2B; see Table S1 at https://doi.org/10.5281/zenodo.16565895). Temperature readings were obtained using a temperature probe. The retrieved samples were centrifuged for 5 min at 6,000 rcf, and the supernatant was discarded. Aliquots were frozen rapidly using liquid nitrogen and kept at −80°C until processing. Samples for SEM were fixed in a solution of 2.5% glutaraldehyde and kept at 4°C until further processing. SEM, elemental composition analysis, and genome-resolved metagenomics using Illumina NovaSeq sequencing were performed as in a previous study (85).

### Genome database of *Gallionella* and Zetaproteobacteria

A genome database of *Gallionella* and Zetaproteobacteria MAGs reconstructed from hydrothermal vents at AMOR and all related publicly available genomes was established. All *Gallionella* genomes based on NCBI taxonomy (taxid 96) and additional *Gallionella* genomes according to GTDB were downloaded with metadata from NCBI Genbank on 116 December 2021. Since *Ferrigenium* (137, 138) and *Gallionella* have been seen as belonging to the same genus cluster (46), *Ferrigenium* genomes were included. IMG search for Gallionellaceae and *Gallionella* revealed 91 items, none connected to marine environments. NCBI GenBank for NCBI taxonomy Gallionellaceae (taxid 90627) revealed 91 MAGs, with most reconstructed from groundwater and freshwater sediment. All publicly accessible Zetaproteobacteria genomes (taxid 580370) and associated metadata were obtained on 10 September 2023 using ncbi-genome-download v.0.3.0 (https://github.com/kblin/ncbi-genome-download/). Additionally, publicly accessible genomes of Zetaproteobacteria were downloaded from the Joint Genome Institute Integrated Microbial Genomes (JGI IMG), Genomes from Earth’s Microbiome database (74), and from public repositories used by selected studies (139–142).

Genomes reaching the quality criteria of more than 50% complete, less than 10% contaminated (143), were used in the subsequent analyses. The assessment of genetic relatedness was carried out using a combination of average nucleotide identity (ANI) and average amino acid identity (AAI). The species cutoff, proposed to be approximately 95% ANI (144, 145), approximately 95–96% AAI (146), with a genus boundary at 65% AAI (147), was employed. The ANI analysis was conducted using the anvi-compute-genome-similarity program within Anvio v.7.0 (148) with the following parameters: --program pyANI --method ANIb (https://github.com/widdowquinn/pyani). The number of unique species-representative genomes was estimated clustering at 95 ANI using the dRep tool (149). AAI analysis was performed using ezAAI (150).

### Phylogenetic and phylogenomic analyses of *Gallionella* and Zetaproteobacteria

Single-copy marker genes were detected and extracted using Anvio v.7.0 (148), utilizing Anvio’s Bacteria_71 and GTDB’s bac_120 collection of single copy marker genes (151). The selection of 15 marker genes for phylogeny of *Gallionella* was based on criteria such as genes being present only in a single copy in *Gallionella* genomes, having a maximum of 2 gene copies in Gallionellaceae MAGs, being found in at least 65% of all *Gallionella* genomes and being found in at least 3 out of 5 marine *Gallionella* MAGs (Table S6). To manually inspect the selection of markers, phylogenetic trees of selected marker gene protein sequences were constructed using ultrafast bootstrapping (152). MAFFT L-INS-i v7.397 (153) was used to create individual marker gene alignments, which were trimmed using trimAl v1.4. rev15 with the -gt 0.5 -cons 60 trimming option (154) and concatenated using catfasta2phyml (https://github.com/nylander/catfasta2phyml). A maximum-likelihood tree was created using IQ-TREE v2.0.3 (155), with SH-like approximate likelihood ratio test (156) and ultrafast bootstrapping (152) with 1,000 iterations, and best-fit model GTR20+F+R6 determined using ModelFinder (157). Phylogenomic analysis of Zetaproteobacteria was done as described in a previous study (85), with an SH-like approximate likelihood ratio test (156) and ultrafast bootstrapping (152) with 1,000 iterations, and best-fit model LG+F+R9 determined using ModelFinder (157).

### Functional annotation, functional enrichment, and wider genomic adaptations

Gene calling and functional annotation of *Gallionella* and Zetaproteobacteria MAGs was performed as described in a previous study (85). Annotation involved an automated pipeline for general annotation (158), a customized script based on KEGG decoder v1.2.1 to identify CO_2_ fixation pathway genes (159, 160). FeGenie v1.1 for iron oxidation genes (161), MagicLamp v1.0 for RubisCO forms and manganese oxidation genes (162), a local BLAST for potential stalk formation gene homologues (99) and BacMet database v2.0 for identifying metal resistance genes (163).

Genus-level pangenome of *Gallionella* and *Mariprofundus* MAGs with >70% completeness and <5% contamination was reconstructed using the anvi-pan-genome script within Anvi'o v.7.0 (148). Parameters were set for –minbit 0.5 –mcl-inflation 2 (164) and for both -min occurrence 2 (genes shared in minimum two MAGs, removing singletons) and -min occurrence 1, choosing the most appropriate pangenome visualization. Construction of the pangenome was based on presence and absence of gene clusters, here defined as gene groups with amino acid sequences of high similarity (165).

Functional enrichment analysis of *Gallionella*, Zetaproteobacteria, and *Mariprofundus* MAGs was performed with MAGs with >70% completeness and <5% contamination using anvi-compute-functional-enrichment within Anvi'o v.7.0 (148) and KEGG, Pfam, and NCBI COG as annotation sources. Functional enrichment analysis identified functions that are characteristic of specific groups and enriched in genomes belonging to one group but largely missing in genomes from other groups, more than expected by chance. The analysis takes into consideration the potential imbalance in the number of observed genomes between different categories. Results were interpreted using the enrichment score (computed with a functional enrichment test using a generalized linear model and logit linkage function), *P* value for each function and *q* value (*P* value adjusted for false detection rate to account for multiple tests). For *q* values less than 0.05, functions were considered enriched (165) and were further investigated.

Interpretation of results of functional enrichment analysis required caution due to inherent biases in genome grouping based on metadata, a limited number of MAGs in some environmental categories, and potential gene content redundancy due to over-representation of some species-representative genomes. Environmental categorization could be influenced by incomplete metadata, especially concerning salinity distinctions. The environments were assigned to MAGs based on available metadata (assigning groundwater as freshwater) and outcomes discussed based on possible salinity differences involved (elevated salt concentrations in groundwater due to mixing with seawater or dissolved minerals in hot springs). Statistical tests might be less reliable for certain environmental categories, such as marine *Gallionella,* which included five MAGs as the sole available ones to date. Although recognizing the potential for overrepresentation of certain traits caused by redundant MAGs, our selection retained all MAGs meeting the above-mentioned quality thresholds to ensure the preservation of crucial genes at risk of loss during dereplication of genomic variability at 95% ANI (166). Addressing these challenges, the analyses were used to pinpoint key genes of interest that were subsequently reviewed across genomes and served as insights to possible adaptations.

Estimated genome size was calculated by considering the genome size (count of all bases in the assembled genome) and its completeness, predicted by CheckM2 v1.0.2. Both one-way ANOVA and Tukey’s HSD post hoc test, and Kruskal-Wallis and post hoc Dunn’s test were performed in base R, while statistical power analyses were performed using the pwr v.1.3-0 R package to determine the necessary sample size per group to obtain a power of 0.80, with various effect sizes considered and a significance level of 0.05. Average isoelectric points (pI) of the predicted proteomes were calculated using IPC 2.1 (167) and analyzed based on a previous study (23).

### Ectoine synthase

The ectoine synthase protein sequences were identified in *Gallionella* MAGs with their closest relatives identified by blastp alignment using GenBank and nr database (October 2023) (168, 169). MAFFT L-INS-i v7.397 (153) was used to create the alignment of the sequences, which was then manually reviewed and trimmed using trimAl v1.4. rev15 with the -gappyout trimming option (154). A maximum-likelihood tree of 177 sequences and 130 positions of comparable length was created using IQ-TREE v2.0.3 (155) with SH-like approximate likelihood ratio test (156) and ultrafast bootstrapping (152) with 1,000 iterations, and best-fit model LG+I+I+R4 determined using ModelFinder (157). The tree was rooted at the midpoint. Environment data and taxonomy were pulled from available metadata from NCBI. Ectoine synthase gene cluster and its gene neighborhood were collected from Anvi'o v.7 (148) using anvi-export-locus. These clusters were re-annotated with Prokka v1.14 (170) to generate new .gbk files which were then analyzed and visualized using clinker v0.0.23 (171).

## Data Availability

All MAGs in the study were deposited in NCBI, and accession numbers with associated BioProject and BioSamples with corresponding metadata are listed in Tables S1 to S4 in the Zenodo repository at https://doi.org/10.5281/zenodo.16565895.
